# Assessing the potential impacts of a changing climate on the distribution of a rabies virus vector

**DOI:** 10.1371/journal.pone.0192887

**Published:** 2018-02-21

**Authors:** Mark A. Hayes, Antoinette J. Piaggio

**Affiliations:** 1 Normandeau Associates Inc., Gainesville, Florida, United States of America; 2 National Wildlife Research Center, U.S. Department of Agriculture, Fort Collins, Colorado, United States of America; Università degli Studi di Napoli Federico II, ITALY

## Abstract

Common vampire bats (*Desmodus rotundus*) occur throughout much of South America to northern México. Vampire bats have not been documented in recent history in the United States, but have been documented within about 50 km of the U.S. state of Texas. Vampire bats feed regularly on the blood of mammals and can transmit rabies virus to native species and livestock, causing impacts on the health of prey. Thus cattle producers, wildlife management agencies, and other stakeholders have expressed concerns about whether vampire bats might spread into the southern United States. On the other hand, concerns about vampire-borne rabies can also result in wanton destruction at bat roosts in areas occupied by vampire bats, but also in areas not known to be occupied by this species. This can in turn negatively affect some bat roosts, populations, and species that are of conservation concern, including vampire bats. To better understand the current and possible future distribution of vampire bats in North America and help mitigate future cattle management problems, we used 7,094 vampire bat occurrence records from North America and species distribution modeling (SDM) to map the potential distribution of vampire bats in North America under current and future climate change scenarios. We analysed and mapped the potential distribution of this species using 5 approaches to species distribution modeling: logistic regression, multivariate adaptive regression splines, boosted regression trees, random forest, and maximum entropy. We then projected these models into 17 “worst-case” future climate scenarios for year 2070 to generate hypotheses about how the vampire bat distribution in North America might change in the future. Of the variables used in this analysis, minimum temperature of the coldest month had the highest variable importance using all 5 SDM approaches. These results suggest two potential near-future routes of vampire bat dispersal into the U.S., one via southern Texas, and a second into southern Florida. Some of our SDM models support the hypothesis that suitable habitat for vampire bats may currently exist in parts of the México–U.S. borderlands, including extreme southern portions of Texas, as well as in southern Florida. However, this analysis also suggests that extensive expansion into the south-eastern and south-western U.S. over the coming ~60 years appears unlikely.

## Introduction

The conservation and management of wildlife species is improved with a robust understanding of geographic areas that provide suitable habitat for a given species of concern [1–4]. The distribution patterns of highly visible species, such as large and diurnal mammals, are usually relatively well-known, but much less is known about the distributions of elusive, nocturnal, or cryptic mammalian species. For example, many species of bat are small, reclusive, and rarely active or easily observable during the daytime. Thus, less is known about the distribution patterns of small, nocturnal bats compared to highly visible and/or gregarious diurnal bats, such as Old World fruit bats (family Pteropodidae). The compilation and analysis of occurrence records in combination with species distribution modeling has been used to address many problems in theoretical and applied ecology, including suggesting sites with high potential for occurrence of rare or cryptic species, and for development of management plans and conservation strategies under current and future climate scenarios [2–4]. Several researchers and policy groups have emphasized the value of using currently-available data and quantitative models, such as species distribution models, to evaluate the relationships between areas currently occupied by species of management concern to other geographic areas that may currently represent suitable habitat for a given species, or might become suitable in the future [5–7]. Recent research has considered the potential expansion and contraction of the distributions of bat species of management and conservation concern under the influence of environmental change [8–13]. For example, recent research suggests that the distributions of Kuhl’s pipistrelle (*Pipistrellus kuhlii*) [9] and Savi’s pipstrelle (*Hypsugo savii*) [10] may be expanding in part due to environmental and climate changes in south and central Europe. Alternatively, some species of management and conservation concern in western North America may be at risk of substantial population declines and contracting distributions as a result of changing climate and water resource conditions [11–13].

Common vampire bats (hereafter “vampire bats”, *Desmodus rotundus*) occur throughout much of South America to northern México [14,15]. Vampire bats feed regularly on the blood of mammals and can transmit rabies virus to prey [16–19], sometimes causing substantial impacts to agricultural economies when rabies virus is transmitted to cattle [20,21]. Vampire bats have not yet been documented in recent history in the United States, but have been documented within about 50 km of the U.S. state of Texas (U.S. Department of Agriculture (USDA) and Méxican Secretariat of Agriculture, Livestock, Rural Development, Fisheries, and Food (SAGARPA); see the data package for this project and methods section). Fossil records indicate that vampire bat species (genus *Desmodus*) existed in what is now the U.S. previously [22], and these fossil records indicate that now-extinct species of vampire bats were in the U.S. from 30,000 years ago until 5,000 years ago. Fossils of different species of vampire bat have been found in the western U.S. from west Texas to northern California and in the eastern U.S. in Florida and West Virginia [22]. Fossils of vampire bats have also been found in Cuba and date to the Holocene [23,24].

Recent research suggests that vampire bats readily exploit a variety of prey resources [25], including livestock and invasive mammals, such as feral pigs [18], and that males in particular can rapidly disperse in response to newly-available prey resources [25]. Although the dispersal distance into the U. S. from currently occupied areas would be relatively short (e.g. from north-eastern México into the southern tip of Texas; < 200 km), some stakeholders are concerned that one or more established populations of vampire bats would represent a novel, invasive colonizer [26], with the potential to have significant impacts on the new environment and the local agricultural economy. Cattle densities are relatively high in north-eastern México—including in the states of Tamaulipas and Nuevo Leon—and in southern Texas and elsewhere in the south-eastern United States [27]. Feral pigs, which are a key food resource for vampire bats in some parts of their range [18,19], are widespread in the south-eastern U.S., including throughout most of Texas [28]. Although the economic impacts of vampire bats are difficult to quantify, they are known to weaken cattle due to loss of blood, lead to secondary infections, reduce milk production, and lead to death if cattle contract paralytic rabies [21,29]. Thus, vampire bats are of concern to livestock industry producers and managers who strive to control the spread of disease among cattle herds. Vampire bats are also of concern to biologists and wildlife managers, due to the destruction of some bat roosts that contain species of conservation concern that were incorrectly thought to contain vampire bats [14,15,17,20].

There are contradictory studies on whether suitable habitat currently exists for vampire bats in the southern U. S., especially in areas with high cattle, livestock, and feral pig densities. Several previous studies have evaluated the potential future North American distributions of vampire bats under the influence of a changing climate. Ceballos et al. [30] suggested that future climate scenarios might result in some parts of the México–U.S. borderlands becoming suitable habitat for vampire bats. Mistry and Moreno-Valdez [31] concluded that parts of the United States could become suitable habitat in the future, including southern Texas, Louisiana, Florida, and perhaps Arizona and California. Alternatively, Lee et al. [32] concluded that vampire bats will “…not expand into the U.S. through Mexico” through the year 2070 (pp7). Most recently, Zarza et al. [33] analyzed the distribution of vampire bats in México and evaluated the potential impacts of a changing climate on the species; their analysis suggests that suitable habitat for vampire bats will not extend into the U.S. under future climate scenarios. However, each of these previous analyses either: did not explicitly extrapolate models into the United States [24,30,33]; used only one species distribution modeling approach (Maxent; [32, 33]); used only one or two future climate scenarios [31–33]; or developed current species distribution models using vampire bat occurrence data from two continents (North and South America; [32]), the latter potentially complicating projections of the models into future climate scenarios in North America.

The purpose of this study was to use multiple species distribution modeling (SDM) approaches (N = 5) to model and map the potential distribution of vampire bats in North America under current and “worst-case” future climate scenarios (N = 17), focusing specifically on the northern limits of the current vampire bat distribution and the México-U.S. borderlands. Our rationale was to develop well-justified and empirically-based hypotheses of the current and future potential distributions of this species, especially in relation to the risk of vampire-borne rabies virus to cattle in the extreme southern United States (such as south Texas). Concerns about vampire-borne rabies can also result in wanton destruction at bat roosts in areas known to be occupied by vampire bats, but also in areas not known to be occupied by this species, which can in turn negatively affect some bat roosts, populations, and species that are of conservation concern [34]. Thus, a key goal of this project was to better understand the current and possible future distributions of vampire bats so that current and future management activities can be targeted most effectively at appropriate vampire populations, but not adversely influence other bat populations and species of conservation concern. This project is part of a broader research program aimed at better understanding the ecology, population genetics, and fine-scale distribution patterns of vampire bats in northern México. In an attempt to capture the possible current and future distributions of vampire bats, we used five SDM approaches that have performed well in comparative SDM analyses [7], including for bats. These approaches include a classical statistical model (logistic regression within the generalized linear models (GLM) and maximum likelihood framework), but also newer machine learning algorithms, such as random forest and maximum entropy approaches. We developed a set of carefully-chosen predictor variables, then pooled the predictions from each SDM model approach and generated ensemble maps; such ensemble methods often provide better overall prediction when compared to single model approaches [35,36]. We included future climate projections from all 9 national climate modeling centers that were used in the most recent IPCC CMIP5 reports (the Intergovernmental Panel on Climate Change’s Coupled Model Intercomparison Project [37]).

## Methods

### Data for modeling and evaluation

**Fig 1:**
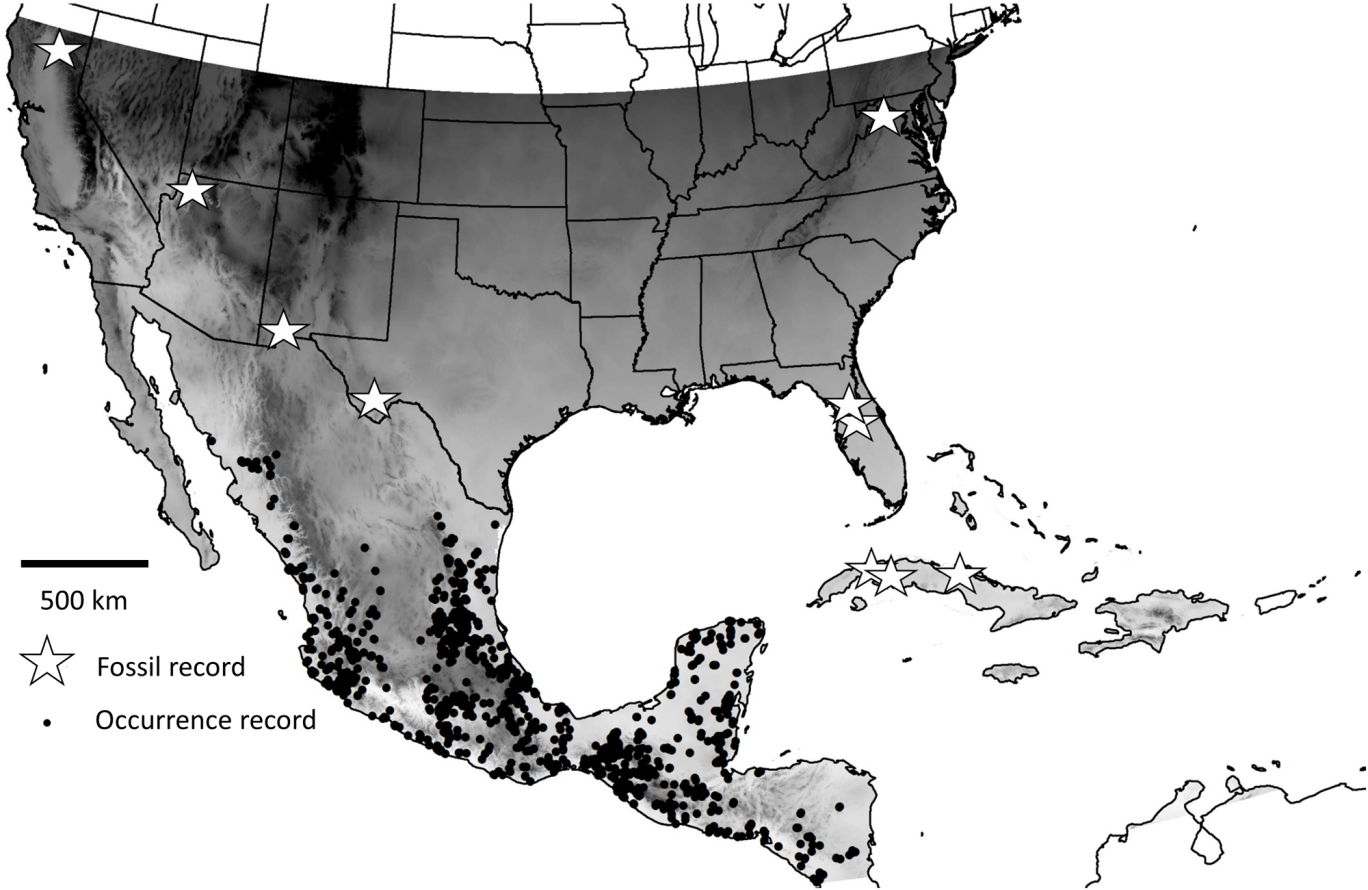
Spatial extent and occurrence record locations of common vampire bats (*Desmodus rotundus*) in North America. The spatial extent is indicated by grey background. Black dots represent 1,029 occupied pixels using 7,094 individual occurrence records for vampire bats in México. The white stars indicate fossil locations of vampire bats (genus *Desmodus*) known from the United States and Cuba.

We used museum and other occurrence records of vampire bats collected in México available in the Global Biodiversity Information Facility dataset (www.gbif.org) and by Méxican collaborators. We added to this data set over 600 records representing 77 vampire bat occurrence locations compiled by Piaggio et al. [38], which were originally compiled for a study investigating vampire bat population genetics in the north-eastern portion of their range. The extent of the analysis (Fig 1) included North America from the southern México border with Guatemala to the latitude of the northern-most location of vampire bat fossils in the genus *Desmodus* (Fig 1; Potter Creek Cave, Shasta County, California, ~40° 47’ N; [22,39]). We used the fossil record for *Desmodus* to define the northern limit of the template because we were interested in including areas that, although not recently inhabited by *Desmodus*, have been inhabited by this genus since the late Pleistocene. The extent was expressed as a raster with cell resolution of 2.5 arc-minutes (~5 km x 5 km) in the North American Albers Equal Area Conic projection (NAD83 datum). We eliminated occurrence records that had information identifying locality to only Méxican states. Background samples were generated by selecting a random sample of 10,000 points from all available cells in the extent [3]. If a background sample coincided in the same cell with an occurrence record, the cell was classified as being occupied. These background points were compared to cells known to be occupied by vampire bats using each of 5 SDM algorithms (see below); thus, each algorithm was used as a binary classifier to predict whether a given cell on the template represented suitable habitat for vampire bats.

We considered the potential problem of sample selection bias [40] and spatial autocorrelation [41] associated with our vampire bat occurrence data, and to evaluate whether sampling bias and spatial autocorrelation was a concern in our dataset we evaluated patterns of distance among occurrence locations and evaluated the spatially-structured variance in our data [42–44]. We used the ‘spatstat’ package [45] for R statistical software [46] for spatial point pattern analysis to compute distances among occurrence locations and to evaluate spatial autocorrelation. We then used this information to consider the distance among unique occurrence locations in our final dataset, patterns of spatial clustering in occurrence records, and whether nearest neighbor occurrence locations were likely to be spatially independent. We concluded that use of thinning or other bias correction procedures (such as target background approaches; [40]) were not necessary given that our purpose was to develop empirically-based hypotheses of the distribution of vampire bats in North America. Furthermore, we concluded that some bias correction procedures, such as target background approaches, might add unknown biases to the modeling dataset, and we concluded that we could not assume that any sampling bias in the vampire bat data was equivalent to the bias in a higher taxonomic group (such as that of the family Phyllostomidae) using easily accessible online data (such as that contained in the Global Biodiversity Information Facility data; www.gbif.org).

The predictor variables considered were selected based on their relevance as plausible predictors of vampire bat occurrence, but we restricted use of predictor variables to those that had a North American or global extent, an estimated resolution of 2.5 arc-minute (~5 km x 5 km), and which could be used in future climate scenarios. As potential predictor variables, we considered use of WorldClim climate data (°C, 1950–2000; [47]), USGS topographic data (elevation (m); [48]), MODIS Vegetation Continuous Field data [49], and MODIS Phenology data [50]. We considered using current prey density as a predictor (e.g., cattle and feral pig densities) but did not have access to projections of how prey density might change under the climate change scenarios used in this analysis. We evaluated the correlation between each possible pair of predictor variables and eliminated one variable from each pair that was strongly correlated (Pearson or Spearman correlation, r > 0.70; [7]). During winter, vampire bats avoid areas with prolonged cold temperatures that would make normal activity excessively challenging, especially given that they are known to have difficulty defending body temperature at low ambient temperatures and do not hibernate [51,52]. McNab [52] used metabolic rates and field observations of behavior to propose that the distribution of the vampire bat was constrained by the 10°C January isotherm. Vampire bats also require daily access to blood meals [52]. Thus, we suspected that vampire bats seek areas with moderate winter temperatures that allowed for continued year-round activity and pursuit of prey resources. Thus, we included minimum temperature of the coldest month (WorldClim Bio6; °C) as a predictor. We also used Worldclim diurnal temperature range data (Bio2; °C) as a predictor, given that vampire bat activity is likely affected by daily temperature variation. Annual precipitation (Bio12; mm) and precipitation of the coldest quarter were also used as predictors, as we expected precipitation to influence the availability of vegetation that might influence prey availability and availability of roosting resources in trees. We considered using remote-sensed phenology and vegetation variables, such as percent tree cover (derived from MODIS Vegetation Continuous Field data; [49]), and vegetation phenology data, such as length of growing season (MODIS Phenology data; [50]). However, we concluded that the addition of such data in future climate models might add additional uncertainly to the final model projections and maps. The final set of predictor variables used in this analysis are shown in Table 1.

**Table 1:** Final set of five predictor variables used in modeling potential habitat suitability and distribution of common vampire bats (*Desmodus rotundus*) in North America under current climate and future climate projections.

Variable Description	Bioclim Code	Units
Mean diurnal temperature range (mean of monthly max temp–monthly min temp)	Bio2	°C
Minimum temperature of the coldest month	Bio6	°C
Annual precipitation	Bio12	mm
Precipitation seasonality	Bio15	Coefficient of variation
Precipitation of coldest quarter	Bio19	mm

For Bio19, a quarter is defined as a period of three months, with the first quarter beginning on January 1.

### Modeling methods and evaluation

We analyzed the potential distribution of vampire bats in México and the southern U.S. using 5 SDM approaches: generalized linear models using logistic regression and maximum likelihood estimation (GLM), multivariate adaptive regression splines (MARS), boosted regression trees (BRT), a random forest algorithm (RF), and maximum entropy (Maxent), using 10-fold cross-validation for each approach [3,4]. We used the Software for Assisted Habitat Modeling (SAHM) package for VisTrails software to fit species distribution models, perform model selection, and calculate performance metrics [53]. For GLM, we used a bidirectional stepwise procedure using Akaike’s Information Criterion (AIC), considering all interactions and squared terms. For MARS, we used Mars Degree (Friedman’s μ) = 1 and GVL penalty = 2.0. For BRT, we used alpha = 1, bag fraction = 0.5, and number of folds = 3, with other values at default values. For RF, we used the tuneRF function to minimize out of bag error. For Maxent, we allowed linear, quadratic, product, threshold, and hinge features, and used regularization values as linear/quadratic/product = 0.050 and hinge = 0.500.

The area under the receiver operating characteristic curve (referred to as AUC) is often used in the analysis of SDM results [3,4]. Sensitivity (a model’s ability to predict true presences) and specificity (a model’s ability to predict background cells) are also commonly used SDM metrics for comparing model performance [54]. We calculated AUC, sensitivity, and specificity to evaluate each SDM’s performance. For threshold-dependent metrics (sensitivity and specificity) we used the threshold that maximized the average of sensitivity and specificity [4]. We evaluated variable importance by SDM approach by using change in the AUC statistic with and without the variable, but with all other variables used; thus, we calculated increase in AUC (ΔAUC) when each predictor variable was permuted for each model approach, and then ranked predictor variables by mean ΔAUC.

Each model produced an estimate of potential habitat suitability for each cell, expressed as continuous values between 0 and 1, given the data, model, and predictor variables used. Using the threshold described above for each SDM approach, we mapped potential habitat suitability for each SDM approach using binary maps. We then generated an ensemble map of potential vampire bat distribution by using the average of the binary estimates (0 or 1) of the 5 models for each cell on the template [36]. We used the SAHM [53] output and R statistical software [46] to create response curves for each of the 5 SDM approaches using the highest-scoring predictor variable.

### Future climate models and maps

For each of the 5 SDMs we projected the final current-climate SDM for vampire bats into future climate projections for North America using the World Research Programme’s most current Coupled Model Intercomparison Project set of future climate projections for year 2070 (CMIP5; [37]), and the RCP8.5 representative concentration pathway emissions scenario and climate projections. The RCP8.5 representative concentration pathway is the potential climate pathway that is expected to result in the most significant changes in climate forcing relative to the climate of the recent past [37]. We used the RCP8.5 projections because this represents the largest change from current climate of the four representative concentration pathway scenarios. Since it is currently unclear which future climate model provides the best predictions for our area of interest (especially the México-U.S. borderlands), we extrapolated each of the 5 final current-climate SDMs for vampire bats into each of the 17 future climate projections derived from global circulation models (RCP8.5 GCMs), representing 9 national climate change research centers produced by climate scientists in 7 nations (Table 2). Each of these future climate models produced a binary estimate of habitat suitability for year 2070 for each cell on our North American template. We then created a grand ensemble future climate map by combining in one map the binary results for each of the 5 SDM results applied to each of the 17 future climate models. This approach combined 85 models (5 SDM approaches x 17 GCM future climate projections) into a final grand ensemble map, based on the average of the binary estimates (0 or 1) for all model runs for each cell on the template. Given that our goal in this project was to consider “worst-case” influences of a changing climate on vampire bat distributions in North America, we decided not to complete the runs required for the other emission pathways, which would have required roughly 17 × 3 = 51 additional dedicated computer days, given the resources we had available at the time of this analysis. A data and code package used in this analysis are available in a GitHub repository (https://github.com/mark-a-hayes/Hayes-Piaggio-2017). This repository includes geographic locations in decimal degrees of the occurrence data used in the analysis.

**Table 2:** List of the CMIP5 climate models used in this study.

Model Name	Abbreviation	Nation	Institution
ACCESS1-0	AC	Australia	Australian Community
BCC-CSM1-1	BC	China	Beijing Normal University
CCSM4	CC	USA	National Center for Atmospheric Research
CNRM-CM5	CN	France	Centre National de Recherches Météorologiques
GFDL-CM3	GF	USA	NOAA/Geophysical Fluid Dynamics Laboratory
GISS-E2-R	GS	USA	NASA/Goddard Institute for Space Studies
HadGEM2-AO	HD	UK	Met Office Hadley Centre
HadGEM2-CC	HG	UK	Met Office Hadley Centre
HadGEM2-ES	HE	UK	Met Office Hadley Centre
INMCM4	IN	Russia	Institute for Numerical Mathematics
IPSL-CM5A-LR	IP	France	Institut Pierre Simon LaPlace
MIROC5	MC	Japan	Japan Agency for Marine Earth Science
MIROC-ESM	MR	Japan	Japan Agency for Marine Earth Science
MIROC-ESM-CHEM	MI	Japan	Japan Agency for Marine Earth Science
MRI-CGCMM3	MG	Japan	Japan Agency for Marine Earth Science
MPI-ESM-LR	MP	Germany	Max Planck Institute
NorESM1-M	NO	Norway	Norwegian Climate Centre

The CMIP5 climate models are the World Research Programme’s most current Coupled Model Intercomparison Project set of future climate projections for year 2070 (CMIP5; cmip-pcmdi.llnl.gov/cmip5/). The RCP8.5 representative concentration pathway emissions scenario and climate projections were used.

## Results

We compiled 7,094 vampire bat records documented in México, which represented 1,029 occupied 5-km^2^ cells (Fig 1). AUC, sensitivity, and specificity statistics for each of the 5 SDM approaches (GLM, MARS, BRT, RF, and Maxent) are shown in Table 2. AUC scores ranged from 0.930–0.961, with little variation in AUC score among model approaches (Table 3). RF received the highest AUC score, and GLM receiving the lowest AUC score. Sensitivity ranged from 0.788–0.977 (Table 3). MARS received the highest sensitivity, and RF received the lowest sensitivity among all SDM models (Table 3). Sensitivities were similar among model approaches, with the exception of RF, which was substantially lower (0.788) than the other SDM approaches. Specificity ranged from 0.793–0.929 (Table 3). RF received the highest specificity among all analyses, and MARS received the lowest specificity among all SDM analyses (Table 3). AUC and sensitivity tended to be > 0.90, while specificity tended to be < 0.90 (Table 3).

**Table 3:** Comparison of area under the curve (AUC), sensitivity, specificity, and average of sensitivity and specificity ((sensitivity + specificity)/2) statistics using test data and the cross-validation mean for each of 5 species distribution model (SDM) approaches using the final set of predictor variables in modeling potential habitat suitability and distribution of common vampire bat (*Desmodus rotundus*) in North America.

SDM Model	AUC	Sensitivity	Specificity	(Sensitivity + Specificity)/2
GLM	0.930	0.950	0.806	0.878
MARS	0.932	**0.977**	0.793	0.885
BRT	0.950	0.900	0.886	**0.893**
RF	**0.961**	0.788	**0.929**	0.859
Maxent	0.938	0.939	0.828	0.884
Mean	0.942	0.911	0.848	0.880

GLM = Generalized Linear Models using logistic regression in a maximum likelihood framework; MARS = Multivariate Adaptive Regression Splines; BRT = Boosted Regression Trees; RF = Random Forest; Maxent = Maxent approach to maximum entropy modeling. High scores are in bold.

**Fig 2:**
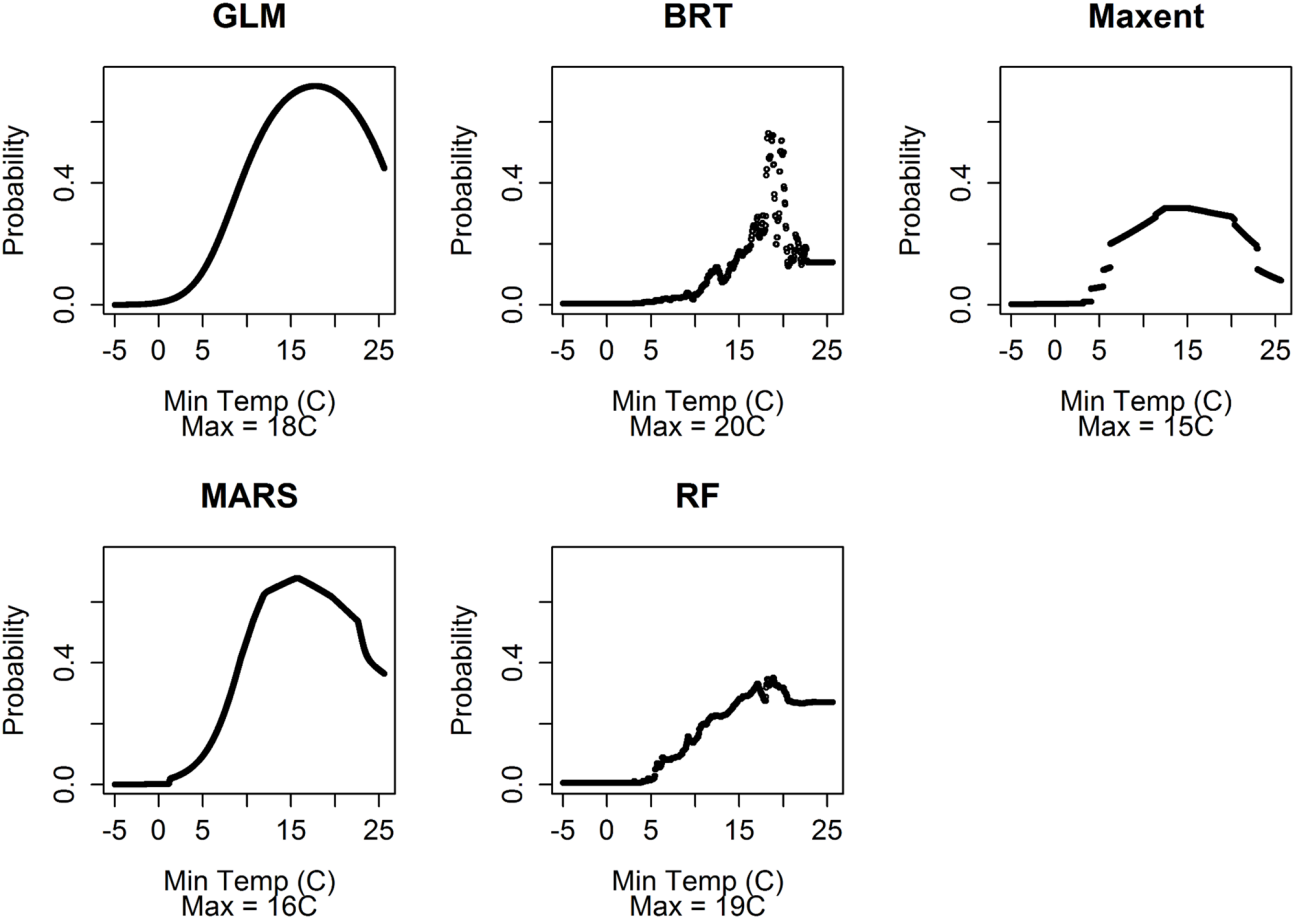
Comparison of response curves for the highest-scoring predictor variable, minimum temperature of the coldest month (Bio6; °C) for each of the 5 species distribution modeling approaches used in this analysis of common vampire bats (*Desmodus rotundus*) in North America. GLM = Generalized Linear Models; MARS = Multivariate Adaptive Regression Splines; BRT = Boosted Regression Trees; RF = Random Forest; and Maxent = Maximum entropy. The x-axis is minimum temperature of the coldest month (Bio6) and the y-axis is the estimated probability that a given value for Bio6 will result in suitable habitat for vampire bats, with all other variables in the model held at their mean values. The “Max” value indicated is the temperature at which the probability is maximized using a given SDM approach.

Variable importance by SDM approach was calculated using increase in AUC (ΔAUC) when each predictor variable was permuted (Table 4). Of the variables used in this analysis, minimum temperature of the coldest month (Bio6; °C) had the highest variable importance using the 5 SDM approaches ($\bar{x}=0.24$, range 0.20–0.37). Precipitation seasonality (Bio15; CV) had the next highest variable importance ($\bar{x}=0.09$, range 0.04–0.12), followed by annual precipitation (Bio12; mm; $\bar{x}=0.06$, range 0.05–0.08), mean diurnal temperature range (Bio2; °C; $\bar{x}=0.02$, range 0.00–0.04), and precipitation of the coldest quarter (Bio19; mm; $\bar{x}=0.01$, range 0.00–0.04). A comparison of the response curves for the highest-scoring variable (minimum temperature of the coldest month; Bio6; °C) is shown in Fig 2. Each of the 5 SDM approaches resulted in a response curve that predicted maximum habitat suitability when minimum temperature of the coldest month (Bio6; °C) is between 15–20°C (Fig 2).

**Table 4:** Variable importance using increase in area under the curve (AUC) statistic when each predictor variable is permuted using 5 species distribution model approaches to model patterns of habitat suitability and distribution of common vampire bats (*Desmodus rotundus*) in North America.

Variable	GLM	MARS	BRT	RF	Maxent	Mean ΔAUC
Minimum temperature of the coldest month (Bio 6; °C)	0.22	0.37	0.23	0.20	0.20	0.24
Precipitation seasonality (Bio 15; coefficient of variation)	0.12	0.04	0.11	0.12	0.08	0.09
Annual precipitation (Bio 12; mm)	0.05	0.05	0.07	0.08	0.07	0.06
Mean diurnal range (Mean of monthly (max tem–min temp)) (Bio 2; °C)	0.02	0.01	0.00	0.04	0.01	0.02
Precipitation of coldest quarter (Bio 19; mm)	0.00	0.01	0.00	0.04	0.01	0.01

GLM = Generalized Linear Models using logistic regression in a maximum likelihood framework; MARS = Multivariate Adaptive Regression Splines; BRT = Boosted Regression Trees; RF = Random Forest; Maxent = Maxent approach to maximum entropy modeling. Minimum temperature of the coldest month (Bio6) had highest variable importance in each of the 5 SDM approaches. Variable order is ranked by mean Δ AUC. For Bio19, a quarter is defined as a period of three months, with the first quarter beginning on 1 January.

**Fig 3:**
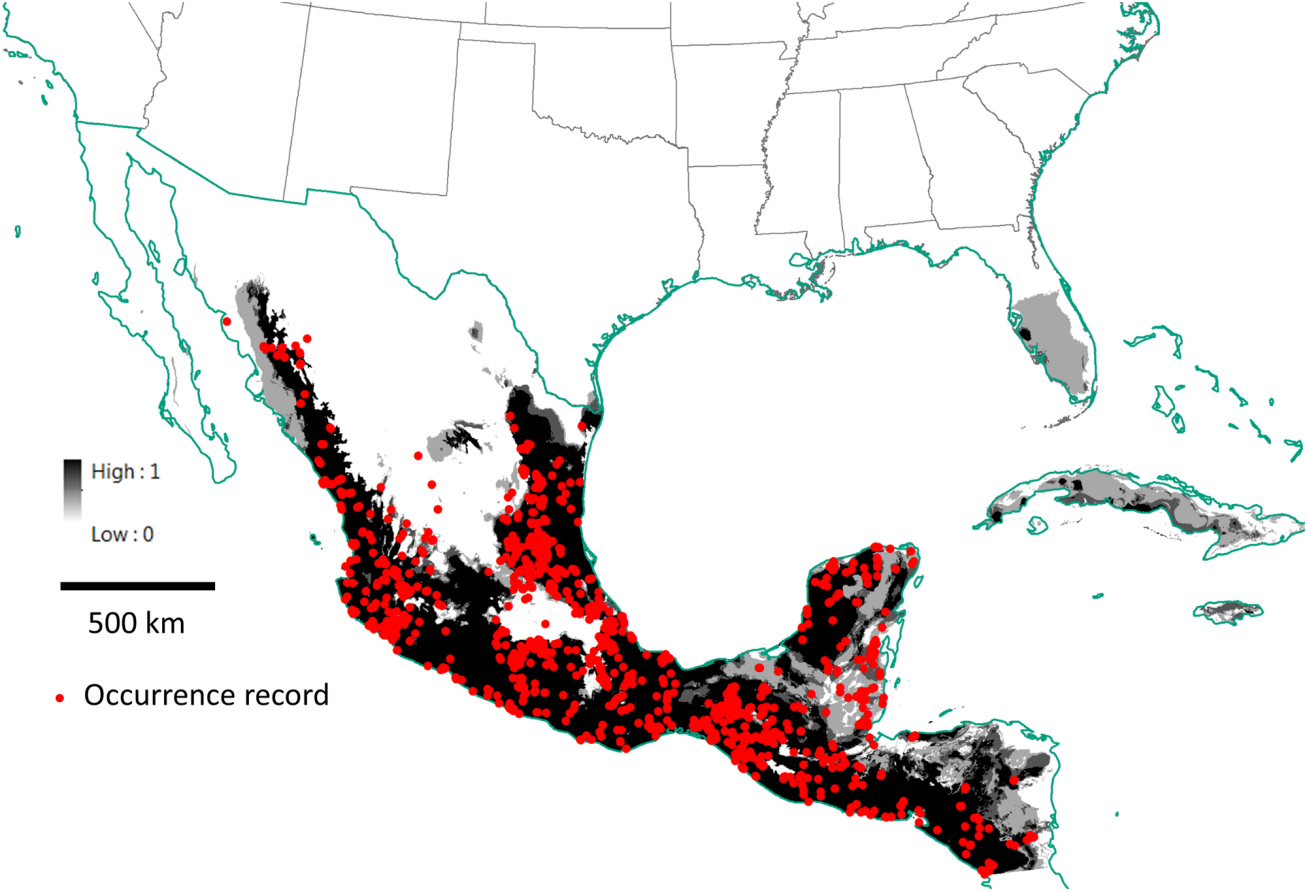
Ensemble map for common vampire bats (*Desmodus rotundus*) in North America using binary prediction from 5 species distribution modeling approaches. Occurrence records are indicated by red dots. The grey-scale indicates the proportion of the 5 model approaches that predict a given cell on the template as suitable habitat. Black indicates that all of the models predict the area to be suitable habitat, while white indicates that all of the models predict the area to be unsuitable habitat.

**Fig 4:**
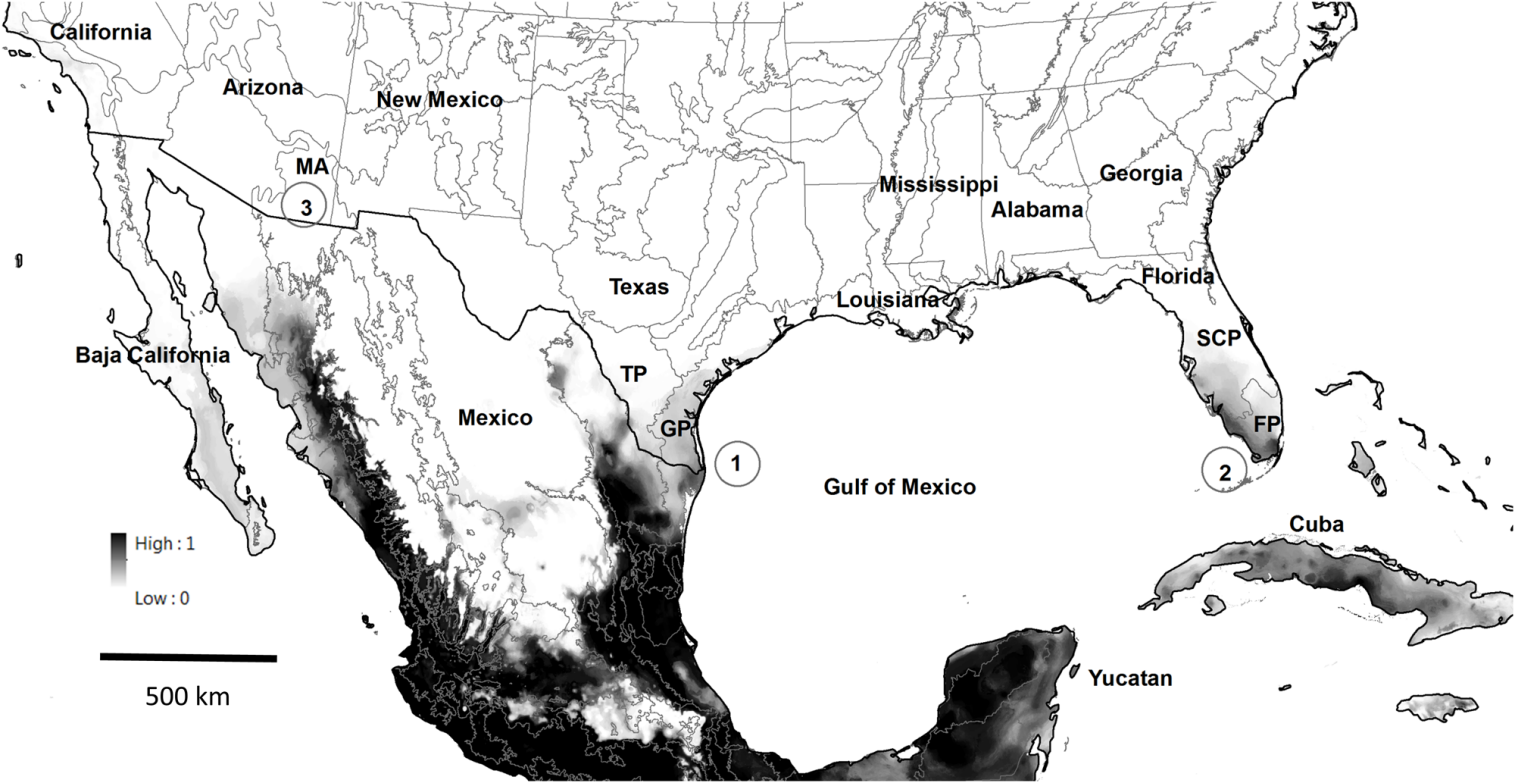
Future climate ensemble map for common vampire bats (*Desmodus rotundus*) in North America using binary predictions from 5 species distribution model approaches projected into 17 downscaled IPPC5 (CMIP5) future climate projections (85 future models; see Table 4) using the 8.5 representative concentration pathway for 2070 (average for 2061–2080). The grey-scale indicates the proportion of the 85 model predictions that considered a given cell on the map as suitable habitat in year 2070. Black indicates that all of the models predict the area will be suitable habitat, while white indicates that all of the models predict the area will be unsuitable habitat. Three possible routes of vampire bat and vampire-borne rabies dispersal into the ecoregions of the United States are also indicated: (1) via the Western Gulf Coastal Plain (GP) and Southern Texas Plains (TP) ecoregions in the México-Texas borderlands; (2) via Cuba and the Southern Florida Coastal Plain (FP) and Southern Coastal Plain (SCP) ecoregions in south Florida; and (3) via the Mandrean Archipelago (MA) ecoregion in New Mexico and Arizona (this route is not likely through year 2070, given the results of this analysis). Outlines of North American ecoregions are indicated, but only ecoregions associated with possible routes of dispersal are named using a 2 or 3 letter acronym.

A current-climate ensemble map using the 5 SDM approaches for common vampire bats in North America is shown in Fig 3. A future-climate grand ensemble map using the 5 SDM approaches for vampire bats in North America is shown in Fig 4; this future-climate map uses the downscaled IPPC5 (CMIP5; [30]) future climate projections using the 8.5 representative concentration pathway for year 2070. Possible routes of vampire bat dispersal into the ecoregions of the United States using the future-climate ensemble map are also shown in Fig 4.

## Discussion

This was the first attempt to map potentially suitable habitats for common vampire bat in North America using multiple SDM approaches focusing explicitly on the México-U.S. borderlands. Our purpose was to use SDMs to evaluate the potential for vampire bats to expand northward into the southern United States. Our results demonstrate how combining species distribution models and ensemble mapping can be a useful tool for developing empirically-based and testable hypotheses of animal distributions and potential future distribution patterns, given the impacts of future climate conditions.

Vampire bats have been observed in diverse habitats throughout much of southern and central México, but do not tend to occur at the higher elevations of the Trans-Méxican Volcanic Belt (including near México City), the Sierra Madre Oriental, and the Sierra Madre Occidental [14,15,55]. These bats are also generally absent from the Chihuahuan Desert and the Sonoran Desert, and are not known to occur on the Baja Peninsula and the Baja California Desert [14]. In northern México, vampire bats occur regularly in the coastal and interior plains and hills east of the Chihuahuan Desert, and at lower elevations of the Sierra Madre Occidental [14,15,55]. Some biologists have noted the similarity in the Méxican habitat occupied by vampire bats and similar habitat in southern portions of the United States [51,56,57]. Other biologists have concluded that southern Texas does not represent suitable habitat for vampire bats [32]. However, given these results it appears possible that some vampire bats occur in the extreme southern portion of Texas and have not yet been detected, or that this species has not yet spread into potentially-suitable habitat, including areas with available prey resources. It is also possible that factors that we did not analyze are preventing the spread of vampire bats into this area. Our results also suggest that the southern half of the Florida Peninsula and parts of Cuba may currently represent suitable habitat for vampire bats (Fig 3).

Lyman and Wimsatt [58] and McNab [52] proposed that the distribution of vampire bats is limited by their poor ability to thermoregulate when exposed to low temperatures, and McNab [51,52] concluded that the distribution of vampire bats is correlated with, and possibly constrained by, the 10°C January average minimum isotherm. Our results support this conclusion that minimum annual temperatures constrain vampire bat distributions. The highest-ranked predictor variable in our analysis was minimum temperature of the coldest month (Bio 6, °C; Table 3), and this variable had substantially more support than any of the other predictor variables used. Additionally, all 5 SDM models predicted maximum habitat suitability between 15–20°C, but decreasing habitat suitability below 5–10°C (Fig 2).

### Influence of future climate change

Some of our SDM models support the hypothesis that suitable habitat for vampire bats may currently exist in parts of the México–U.S. borderlands, including extreme southern portions of Texas, as well as in southern Florida and Cuba (Fig 3). However, our analysis suggests that extensive expansion into the south-eastern and south-western U.S. over the coming ~60 years is unlikely, even under worst-case climate change scenarios (Fig 4). There was not a consensus among SDM approaches about whether suitable habitat for vampire bats will expand into the U.S. GLM and MARS indicate that there might be a slight expansion of suitable habitat into the U.S., especially into southern Texas; BRT, RF and Maxent suggest possible contraction of suitable habitat. Of the SDM approaches used, MARS suggested the largest expansion of habitat northward, while Maxent suggested the most contraction of suitable habitat. There is also substantial uncertainty regarding which global circulation model (GCM) to use for projecting into future climate scenarios, focusing especially on southern Texas, and it is still unclear which of the many GCMs might perform best in this region of the U.S. and the México-U.S. borderlands.

These results suggest two potential future routes of vampire bat dispersal into the U.S., one via southern Texas, and a second into southern Florida. Vampire bats are currently known to occur in two ecoregions of north-eastern México that extend into southern Texas: the Western Gulf Coastal Plains, and the Southern Texas Plains and Hills ([59]; Fig 4). Both of these ecoregions offer relatively contiguous habitat between north-eastern México and southern Texas, consisting of similar geology and vegetation structure. Two of our future climate models (MARS and GLM) suggest that the coastal plains habitat in southern Texas may become suitable in the future, and the MARS model projects suitable coastal habitat to just south of Galveston, Texas by year 2070. Although vampire bats are not strong dispersers, homing experiments suggest that they may be familiar with large expanses of habitat around their seasonal roost sites. For example, they may travel up to 20 km away from their day roosts during nightly foraging bouts [14,59–61], and have been documented traveling 120 km over two days in homing experiments [61].

In 1967, one hairy-legged vampire bat (*Diphylla ecaudata*) was documented in an abandoned tunnel near Comstock, Texas [62,63]. This record is approximately 700 km north of the next most northerly record of this species [63], and may represent an anomalous record a long distance from regularly occupied habitat [64] or may represent an as yet unidentified population in the México-Texas borderlands [62]. Nevertheless, this record indicates that bats in the subfamily *Desmodontinae* (*Desmodus rotundus*, *Diphylla ecaudata*, and *Diaemus youngi*; [64,65]) may occasionally be found and perhaps establish small and undetected populations north of their regularly occupied habitat. Given that recent population genetics data suggests that the vampire bat populations in north-eastern México are expanding spatially and demographically [38], that vampire bats have been recently documented within 50 km of the Texas border, and that there is a contiguous corridor of potentially-suitable habitat between north-eastern México and southern Texas [47], it would not be unreasonable to hypothesize that the southern tip of Texas could become occupied by vampire bats in the future.

Another potential route of dispersal into the U.S. could be into southern Florida either via Cuba or by airline and marine transportation channels that connect currently occupied vampire bat habitat with southern Florida. Vampire bats do not currently occur on Cuba, but our analysis (Fig 3) and another [32] suggests that Cuba may currently represent suitable habitat for vampire bats. It does not seem likely that vampire bats would travel the long distance over water between México’s Yucatan Peninsula and Cuba, and then to Florida, given the long distances between these land masses (ca. 175–200 km). However, it is possible that hurricanes and/or other human mediated events could result in a small number of colonizers becoming established on Cuba, which could serve as a stepping stone to Florida, or being directly transported to southern Florida. Current and future climate models suggest that vampire bats would likely find southern Florida to provide suitable habitat (Figs 3 and 4), potentially resulting in an incipient population.

A third route of potential dispersal into the U.S. over the long term—but not likely through year 2070—is from México via the Madrean Archipelago (also known as the Mandrean Sky Islands), which provides a connection between the Méxican states of Chihuahua and Sonora with Arizona and New Mexico [66]. Vampire bats are not known to occur in the Madrean Archipelago, but this ecoregion is adjacent to the hills and canyons of Sinaloa and Sonora, and could provide a corridor for dispersal into the U.S. if a changing climate resulted in the Mandrean Archipelago becoming suitable habitat for vampire bats at some point in the future. However, our analysis suggests that the Mandrean Archipelago is not likely to represent suitable habitat for vampire bats over the coming ~60 years.

Species of *Desmodus* previously occupied portions of what is now the United States. The fossil record suggests that extinct and extant species of the genus *Desmodus* occurred commonly in some areas of the U.S. as recently as 5,000–35,000 years before present. Fossil vampire bats are known from as far north as northern California (late Pleistocene; [22,39]), and are known from the Big Bend area of western Texas (possibly late Pleistocene; [22]), from the present-day Mandrean Archipelago region of New Mexico (estimated as 29,000–36,000 years before present; [22]), from Cuba (<10,000 years BP; [23]), and from several sites in Florida, as well as from other sites in the U.S. [22]. Thus, it would not be surprising if vampire bats again spread into the U.S. if future habitat and climate conditions became suitable to sustain viable populations.

### Comparing these results to other research

We restricted our analysis to distributional data on vampire bats from North America. Lee et al. [32] used occurrence data from all available records of common vampire bats in North and South America. It is possible that the extent we used for our analyses (North America only) may explain some of the difference in our results. We considered highly plausible the McNab [51,52] hypothesis that minimum annual temperature constrains the distribution of vampire bats, and thus we developed our final set of predictor variables beginning with the minimum temperature of the coldest month (WorldClim Bio 6) predictor. We then selected subsequent predictors that were both biologically plausible and not highly correlated. Mistry and Moreno-Valdez [31] used GCM data to evaluate how the 10°C January isotherm might change in and near the México–U.S. borderlands under future climate change scenarios. These researchers concluded that vampire bats “…probably will expand significantly along the east and west coasts of Mexico and into the southern tip and Gulf Coast of Texas, possibly including lower Louisiana” (pp. 10–11); they also concluded that some areas of Florida, Arizona, California, and the Baja Peninsula could become suitable habitat for vampire bats. We agree with Mistry and Moreno-Valdez that the Gulf coast of southern Texas is the area most likely to become suitable to vampire bats in future years. However, while we conclude that the southern tip of Texas and Florida may become suitable by year 2070, none of our models predicted that Louisiana could become suitable habitat by that time, or that contiguous areas would become suitable between the current northern limit of vampire bats and Arizona or California. Nevertheless, given that prey densities are relatively high in north-eastern México and in southern Texas [27], it would be reasonable to conclude that vampire bats could spread into extreme southern Texas under current and future climate conditions.
